# Use of Body Mass Index and Percentage Overweight Cutoffs to Screen Japanese Children and Adolescents for Obesity-Related Risk Factors

**DOI:** 10.2188/jea.JE20090036

**Published:** 2010-01-05

**Authors:** Masayuki Okuda, Shinichi Sugiyama, Ichiro Kunitsugu, Yuji Hinoda, Yumi Okuda, Komei Shirabe, Norikazu Yoshitake, Tatsuya Hobara

**Affiliations:** 1 Department of Environmental Medicine, Graduate School of Science and Engineering, Yamaguchi University, Ube, Yamaguchi, Japan; 2 Departments of Public Health and Laboratory Medicine, Graduate School of Medicine, Yamaguchi University, Ube, Yamaguchi, Japan; 3 Department of Pediatrics, Yamaguchi-Ube Medical Center, Ube, Yamaguchi, Japan; 4 Yamaguchi Prefectural Institute of Public Health and Environment, Yamaguchi, Japan

**Keywords:** body mass index, Japanese, risk factors, percentage overweight, ROC curve

## Abstract

**Background:**

Cutoffs based on percentage overweight (POW) are used for screening students in Japan; however, body mass index (BMI) is more common in the rest of the world. To screen for risk factors related to obesity among Japanese primary and secondary school students, we compared fasting and postprandial values, and the receiver operating characteristic (ROC) curves for the POW and BMI criteria.

**Methods:**

The subjects were students aged 10 and 13 years living in Shunan City, Japan between 2006 and 2008 (*n* = 6566). POW and International Obesity Taskforce (IOTF) BMI criteria were used to screen for obesity-related risk factors. The lower (20%, 18-year-old equivalent: 25 kg/m^2^) and higher (50%, 18-year-old equivalent: 30 kg/m^2^) cutoffs were examined, and ROC curves were drawn.

**Results:**

Fasting cholesterol levels were higher than postprandial levels. The prevalences of overweight/obesity were 6.6% to 10.0% using the lower cutoff and 0.6% to 5.0% using the higher cutoff. Among overweight subjects under fasting conditions, dyslipidemia was present in 12% to 52%, hypertriglyceridemia in 29% to 54%, hyperglycemia in 11% to 21%, and hypertension in 15% to 40%. Although the use of the lower and higher POW cutoffs resulted in lower sensitivity and the higher specificity, the POW and BMI ROC curves largely overlapped. However, for girls aged 10 years, the POW curve for ≥3 risks factors was lower than that of the latter (*P* = 0.013).

**Conclusions:**

For Japanese aged 10 and 13 years, both BMI and POW are useful for risk factor screening. However, subjects may be misclassified with dyscholesterolemia if postprandial blood samples are used.

## INTRODUCTION

In children and adolescents, obesity is a risk factor for dyslipidemia and high blood pressure,^1^^–^^4^ and results in atherosclerotic changes and risk factor clustering among adolescents.^5^^–^^8^ The increasing prevalence of overweight and obese children is therefore a serious public health concern^9^ and screening tools are required to identify risk factor clustering and adult obesity-related diseases in children.

Across the world, different cutoffs are used to identify overweight children and adolescents. In Japan, the Committee for Research of Appropriate Body Build in Children has recommended that obesity be defined in terms of percentage overweight (POW), which is calculated on the basis of age- and sex-specific body-weight standards in relation to height.^10^ For school-aged children, obesity is defined as a POW of 20% or more. Recently, the Childhood Obesity Working Group of the International Obesity Taskforce (IOTF) proposed an international BMI reference standard (IOTF-BMI) for defining overweight/obesity among children and adolescents^11^^,^^12^; this standard was designed in consideration of data obtained from Asian populations. The proposed cutoffs are based on health-related definitions of overweight (≥25 kg/m^2^) and obesity (≥30 kg/m^2^) for adults, but are adjusted for sex in specific age groups of children. In screening for obesity-related health status in Japan,^10^ overweight is defined as a POW of 20% or more.^1^ However, it remains unclear which overweight/obesity definitions are appropriate for screening Japanese children and adolescents with risk factors.

In the present study, we investigated the receiver operating characteristic (ROC) curves of POW and BMI to identify the best screening criteria for classifying weight status and identifying risk factors among Japanese aged 10 and 13 years. In addition, before biochemical parameters and blood pressure were investigated as risk factors, we compared fasting and postprandial values, because the drawing of blood samples is not limited to fasting conditions in the Japanese school health system.^13^

## METHODS

### Study population

This cross-sectional study, which was conducted from 2006 through 2008 as part of the ongoing Shunan Child Cohort Study, involved fifth-year primary school students (mean age, 10.5 years; range, 10–11 years) and second-year secondary school students (mean age 13.5; range, 13–14 years) in Shunan City, Japan. The baseline data used in this study were obtained from a healthcare program survey, the Shunan Healthy Diet for Children, which is described elsewhere (Okuda M., in press). The surveys comprised anthropometrics, questionnaires, blood tests, and body measurements. The questionnaires contained items related to health status and lifestyle. The present study was approved by the Institutional Review Board of Yamaguchi University Hospital and by the Educational Board and Health and Welfare Department of Shunan City. Written informed consent was obtained from both the students and their guardians. All data were anonymized and collected from the Educational Board.

### Anthropometrics

The height and body weight of all participants were measured from April through June by school nurses during annual medical checkups designed to monitor student health status, in accordance with the Japanese School Health Law. Height was measured to the nearest 0.1 cm while the students stood barefooted, and body weight was measured to the nearest 0.1 kg while the students wore light clothing and no footwear. BMI was calculated as body weight (kg)/height (m)^2^. We classified the students as overweight and obese according to 2 definitions (Table 1). In the IOTF-BMI definition, the cutoffs were based on the percentiles that corresponded to a BMI of 25 in the case of overweight (lower cutoff) and a BMI of 30 in the case of obesity (higher cutoff), for a person aged 18 years. The POW definition was based on the percentage of a reference weight that is based on age, sex, and height.^14^ In obesity-related screening of an individual’s health status, a POW of 20% or more is equivalent to “obesity” according to some definitions and to “overweight” according to others. In Japan, a POW of 50% or more is regarded as “obesity disease,” which requires medical intervention. In the present study, we set 20% as the lower POW cutoff and 50% as the higher cutoff.

**Table 1.** Definitions of cutoffs

	Age (years)	POW (%)	IOTF-BMI (kg/m^2^)
Lower cutoff		Obesity	Overweight, 25 kg/m^2^
Female	10	20	20.29
	13	20	22.98
Male	10	20	20.20
	13	20	22.27

Higher cutoff		Obesity disease	Overweight, 30 kg/m^2^
Female	10	50	24.77
	13	50	28.20
Male	10	50	24.57
	13	50	27.25

POW, percentage overweight (Asayama, 2003).

IOTF-BMI, International Obesity Taskforce definition, using the BMI of an 18-year-old as reference (Cole, 2000); values are age-adjusted BMIs.

### Blood sampling and instrumentation

Blood was collected annually between the months of May and July. Although we recommended a minimum of 10 hours of fasting before morning blood sampling, not all schools mandated overnight fasting. We checked whether the participants ate breakfast on the day of blood sampling. The serum levels of total cholesterol, low-density lipoprotein (LDL) cholesterol, high-density lipoprotein (HDL) cholesterol, and triglycerides were analyzed using an automatic clinical analyzer (Hitachi 7600-110S; Hitachi High Technology Corp., Tokyo, Japan). Plasma glucose levels were measured using the Glucoroder-NX automatic glucose analyzer (A&T Corp., Yokohama, Japan). Blood pressure was measured using an automatic monitor (HEM707, HEM757, or HEM780; Omron Corp., Kyoto, Japan) while the subject was seated, after a 5-minute rest period.

### Definitions of risk factors

Because clear definitions of the risk factors related to obesity in children have not been established, we regarded a subject’s health status as abnormal based on definitions that have previously been used for pediatric populations.^15^^–^^17^ For 10-year-old girls, 13-year-old girls, 10-year-old boys, and 13-year-old boys, dyscholesterolemia was defined as a total cholesterol level of at least 5.04, 5.02, 5.02, and 4.80 mmol/L (195, 194, 194, and 185.5 mg/dL), respectively, an LDL cholesterol level of at least 2.92, 2.90, 2.90, and 2.66 mmol/L (113, 112, 112, and 103 mg/dL), respectively (ie, equaling or exceeding the 80th percentile), or an HDL cholesterol level equal to or lower than the 20th percentile, ie, 1.47, 1.47, 1.53, and 1.47 mmol/L (57, 57, 59, and 57 mg/dL). Hypertriglyceridemia was defined as a triglyceride level equaling or exceeding the 80th percentile, ie, 0.90, 0.90, 0.82, and 0.83 mmol/L (80, 80, 73, and 74 mg/dL); hyperglycemia was defined as a plasma glucose level that equaled or exceeded the 90th percentile, ie, 5.44, 5.38, 5.50, and 5.50 mmol/L (98, 97, 99, and 99 mg/dL); and hypertension was defined as a systolic blood pressure (SBP; 121, 126, 121, and 132 mm Hg) or a diastolic blood pressure (DBP; 77, 80, 77, and 78 mm Hg) that equaled or exceeded the 90th percentile. All the percentiles were derived from fasting conditions. For sensitivity analysis, the 90th and 10th percentile values were used instead of the 80th and 20th percentile values, respectively. In addition, we used cutoff values that have been proposed for the screening of diseases of obesity.^10^

### Statistical analysis

The measured values among the age groups and between fasting and nonfasting conditions were compared using the *t*-test. A trend test (Cochran-Armitage test) was used to compare the frequencies of overweight/obesity risk factors. The sensitivity and specificity for risk clustering (range, 1–4 risks) were calculated for the different definitions. The areas under the curve (AUCs) were compared between the receiver operating characteristic (ROC) curves for BMI and POW using a paired nonparametric approach (Hanley and McNeil method). Statistical analysis was performed using the SAS ver. 9.1 software (SAS Institute Japan Ltd., Tokyo, Japan), and significance was set at *P* < 0.05.

## RESULTS

Among the 10-year-old (4248) and 13-year-old (4101) student cohorts, data from 1607 subjects were excluded due to anomalies in blood testing (*n* = 1586), blood pressure measurement (*n* = 1534), and/or anthropometric testing (*n* = 317). In addition, health status was unknown for 156 subjects, and dyslipidemia, diabetes, or hypertension had been previously diagnosed in 20 subjects. Thus, data for 6566 subjects (3383 10-year-olds; 3183 13-year-olds) were available for analysis (Table 2). As compared to postprandial cholesterol levels, fasting cholesterol levels were higher among the girls and the 13-year-old boys; the mean difference in total cholesterol level was 0.09 to 0.18 mmol/L (3.4–7.1 mg/dL); the mean difference in LDL cholesterol level was 0.10 to 0.14 mmol/L (3.8–5.4 mg/dL) (*P* < 0.01 and *P* < 0.001, respectively). The mean difference in HDL cholesterol level among boys was 0.04 mmol/L (1.5 mg/dL; *P* = 0.036) for 10-year-olds and 0.11 mmol/L (4.2 mg/dL; *P* < 0.001) for 13-year-olds. Regarding dyscholesterolemia among nonfasting subjects, the following prevalences were noted: 13.8% to 18.8% due to total cholesterol, 14.2% to 17.8% due to LDL cholesterol, and 22.3% to 33.3% due to HDL cholesterol. The frequency of hyperglycemia was 12.2% to 14.6%. For the subsequent analyses, we used 3812 fasting blood samples (58.1% of the subjects).

**Table 2.** Characteristics of the subjects

	10-year-old Subjects			13-year-old Subjects		
		
	Fasting	Nonfasting	*P*	Fasting	Nonfasting	*P*
Girls	*n* = 713	*n* = 932		*n* = 1116	*n* = 404	
Height (cm)	139.3 ± 6.8	139.7 ± 6.6	0.151	154.5 ± 5.2	154.6 ± 5.6	0.779
Weight (kg)	33.6 ± 7.1	33.6 ± 6.4	0.961	46.9 ± 7.5	46 ± 7.4	0.039
BMI (kg/m^2^)	17.2 ± 2.6	17.1 ± 2.3	0.458	19.6 ± 2.8	19.2 ± 2.6	0.009
POW (%)	−0.1 ± 13.7	−1 ± 12.2	0.168	−0.1 ± 14	−2.2 ± 13.1	0.009
Total cholesterol (mmol/L)	4.5 ± 0.7	4.4 ± 0.6	0.008	4.5 ± 0.7	4.4 ± 0.6	<0.001
LDL cholesterol (mmol/L)	2.5 ± 0.6	2.4 ± 0.5	<0.001	2.5 ± 0.6	2.3 ± 0.5	<0.001
HDL cholesterol (mmol/L)	1.8 ± 0.4	1.7 ± 0.4	0.057	1.8 ± 0.4	1.8 ± 0.3	0.240
Triglyceride (mmol/L)	0.7 ± 0.4	0.8 ± 0.5	<0.001	0.7 ± 0.3	0.8 ± 0.4	<0.001
Plasma glucose (mmol/L)	5 ± 0.3	5 ± 0.4	0.743	5 ± 0.3	5 ± 0.4	0.136
Systolic blood pressure (mm Hg)	106.2 ± 11.9	106.2 ± 12.2	0.854	106.2 ± 11.2	112.2 ± 10.6	0.659
Diastolic blood pressure (mm Hg)	64.4 ± 9.2	63.6 ± 9.5	0.087	68.8 ± 8.6	68 ± 9.5	0.155

Boys	*n* = 798	*n* = 940		*n* = 1185	*n* = 478	
Height (cm)	138.6 ± 6.2	138.5 ± 5.8	0.806	158.7 ± 7.9	159.8 ± 7.5	0.005
Weight (kg)	34 ± 7.6	33.6 ± 6.7	0.235	48.2 ± 9.9	48.7 ± 8.9	0.293
BMI (kg/m^2^)	17.6 ± 2.9	17.4 ± 2.6	0.206	19 ± 2.8	19 ± 2.6	0.817
POW (%)	0.6 ± 14.9	−0.3 ± 13.7	0.184	0.4 ± 13.9	−0.3 ± 13.3	0.351
Total cholesterol (mmol/L)	4.5 ± 0.7	4.5 ± 0.7	0.426	4.3 ± 0.7	4.1 ± 0.6	<0.001
LDL cholesterol (mmol/L)	2.5 ± 0.6	2.4 ± 0.6	0.099	2.3 ± 0.5	2.2 ± 0.5	<0.001
HDL cholesterol (mmol/L)	1.9 ± 0.4	1.8 ± 0.4	0.036	1.8 ± 0.4	1.7 ± 0.4	<0.001
Triglycerides (mmol/L)	0.6 ± 0.4	0.8 ± 0.5	<0.001	0.6 ± 0.3	0.8 ± 0.4	<0.001
Plasma glucose (mmol/L)	5.1 ± 0.3	5.1 ± 0.4	0.528	5.1 ± 0.3	5.1 ± 0.4	0.414
Systolic blood pressure (mm Hg)	106.2 ± 12.1	105.3 ± 12	0.096	116.6 ± 11.8	115.8 ± 10.9	0.247
Diastolic blood pressure (mm Hg)	64.5 ± 9.8	62.9 ± 10	0.001	66.9 ± 9	65.8 ± 8.6	0.027

BMI, body mass index; POW, percentage overweight; LDL, low-density lipoprotein; HDL, high-density lipoprotein.

The fasting and nonfasting values were compared using the *t*-test. SI unit conversion: total and LDL cholesterol (mg/dl) × 0.025 86 = (mmol/L), HDL cholesterol (mg/dl) × 0.025 87 = (mmol/L), triglyceride (mg/dl) × 0.011 20 = (mmol/L), and plasma glucose (mg/dl) × 0.055 51 = (mmol/L).

We found that the percentages of overweight/obese subjects defined according to the lower POW cutoff were lower (8.7% for 10-year-old girls, 8.7% for 13-year-old girls, 10.3% for 10-year-old boys, and 7.6% for 13-year-old boys) than the percentages derived using the IOTF-BMI definition (15.0% for 10-year-old girls, 15.1% for 13-year-old girls, 15.0% for 10-year-old boys, and 10.0% for 13-year-old boys). The prevalences derived using the higher POW cutoffs were lower (0.7% for 10-year-old girls, 0.6% for 13-year-old girls, 0.6% for 10-year-old boys, and 1.0% for 13-year-old boys) than those obtained using the IOTF-BMI definition (5.0% for 10-year-old girls, 5.0% for 13-year-old girls, 5.0% for 10-year-old male, 1.7% for 13-year-old male).

In general, the overweight/obese subjects tended to have more risk factors (trend test, *P* < 0.05) (Table 3). However, no significant trends were noted among the girls with regard to dyscholesterolemia due to total cholesterol or LDL cholesterol, except among 10-year-old girls with regard to the IOTF-BMI cutoffs and dyscholesterolemia due to LDL cholesterol. There were no significant trends among the boys or 10-year-old girls with regard to hyperglycemia.

**Table 3.** Risk frequencies (%) for the 2 definitions of overweight/obese

		POW				IOTF-BMI			
		
		<20%	≥20%	≥50%		<18 kg/m^2^	≥18 kg/m^2^	≥25 kg/m^2^	
10-year-old girls	(*n* = 713)	(651)	(57)	(5)		(606)	(71)	(36)	
Total cholesterol (≥80th)	(145)	21	12	40	0.438	21	13	18	0.205
LDL cholesterol (≥80th)	(147)	20	28	40	0.069	20	28	36	0.032
HDL cholesterol (≤20th)	(157)	19	51	60	<0.001	18	52	64	<0.001
Triglycerides (≥80th)	(148)	19	44	40	<0.001	18	43	45	<0.001
Plasma glucose (≥90th)	(79)	10	18	20	0.083	10	21	9	0.050
Hypertension (SBP, DBP ≥90th)	(125)	15	39	60	<0.001	14	40	55	<0.001

13-year-old girls	(1116)	(1019)	(90)	(7)		(948)	(112)	(56)	
Total cholesterol (≥80th)	(227)	21	16	29	0.452	21	17	20	0.466
LDL cholesterol (≥80th)	(226)	20	21	43	0.346	20	24	30	0.159
HDL cholesterol (≤20th)	(239)	19	41	71	<0.001	19	38	60	<0.001
Triglycerides (≥80th)	(232)	20	31	14	0.047	20	29	10	0.147
Plasma glucose (≥90th)	(117)	10	17	29	0.011	10	16	30	0.006
Hypertension (SBP, DBP ≥90th)	(197)	16	37	57	<0.001	15	37	60	<0.001

10-year-old boys	(798)	(716)	(77)	(5)		(678)	(80)	(40)	
Total cholesterol (≥80th)	(162)	19	26	60	0.027	19	28	29	0.033
LDL cholesterol (≥80th)	(162)	18	36	60	<0.001	18	33	39	<0.001
HDL cholesterol (≤20th)	(160)	17	44	80	<0.001	16	36	68	<0.001
Triglycerides (≥80th)	(165)	18	44	60	<0.001	17	37	54	<0.001
Plasma glucose (≥90th)	(84)	10	14	20	0.181	10	13	14	0.254
Hypertension (SBP, DBP ≥90th)	(141)	15	40	80	<0.001	14	32	50	<0.001

13-year-old boys	(1185)	(1095)	(78)	(12)		(1067)	(98)	(20)	
Total cholesterol (≥80th)	(237)	19	33	58	<0.001	20	19	27	0.001
LDL cholesterol (≥80th)	(251)	19	45	75	<0.001	21	19	36	<0.001
HDL cholesterol (≤20th)	(261)	20	41	83	<0.001	22	19	42	<0.001
Triglycerides (≥80th)	(245)	18	54	67	<0.001	21	18	43	<0.001
Plasma glucose (≥90th)	(132)	11	13	0	0.689	11	11	11	0.554
Hypertension (SBP, DBP ≥90th)	(208)	15	40	75	<0.001	18	15	39	<0.001

POW, percentage overweight; IOTF-BMI, International Obesity Taskforce definition, using the BMI of an 18-year-old as reference.

The trends resulting from the use of the 2 systems were tested using the Cochran-Armitage test.

**Figure 1.**
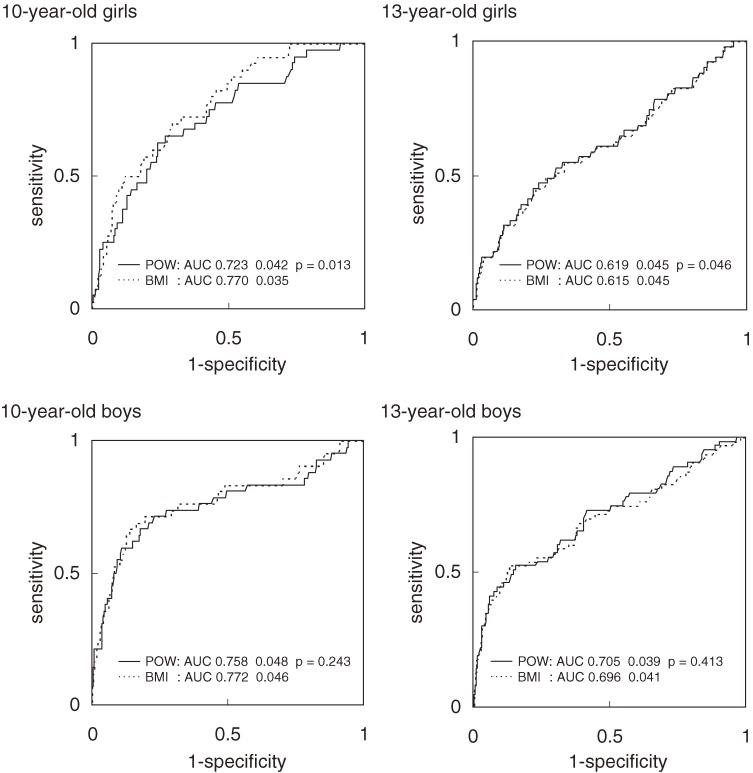
Receiver operating characteristic curves for subjects with 3 or more risk factors. The solid lines represent the POW curves and the dashed lines represent the BMI curves. The AUCs (areas under the curve) were compared using paired nonparametric methods

**Figure 2.**
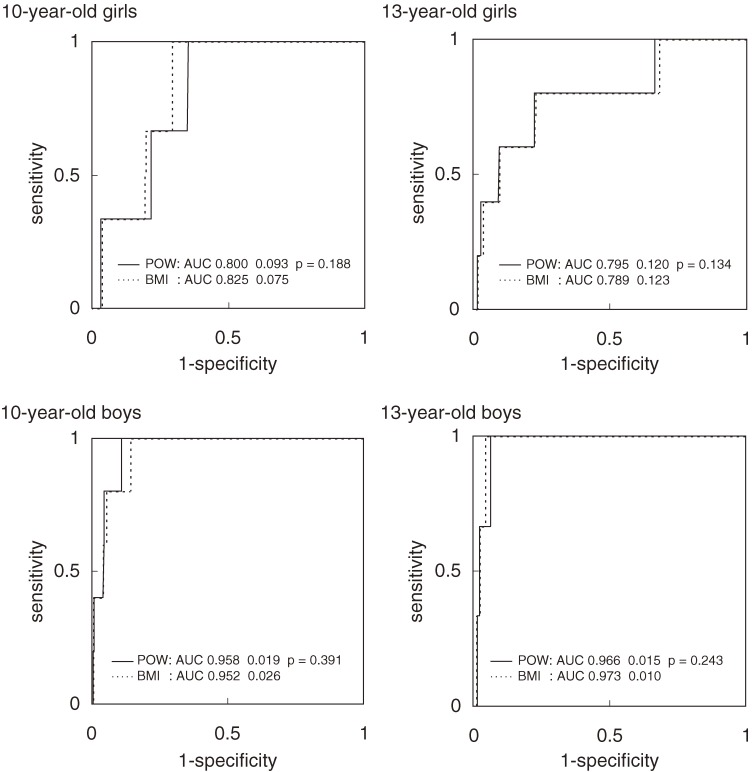
Receiver operating characteristic curves for subjects with 4 risk factors. The solid lines represent the POW curves and the dashed lines represent the BMI curves. The AUCs (areas under the curve) were compared using paired nonparametric methods

The sensitivity for risk clustering (≥3 risk factors) varied with the overweight/obesity definition used (Table 4). The POW-based definition had lower sensitivity (13% for 1 or more risks to 63% for 4 risks with the lower cutoff, and 1%–6% with the higher cutoff), as compared to the IOTF-BMI definition (17%–75% with the lower cutoff, and 3%–19% with the higher cutoff). The POW definition was more specific than the IOTF-BMI definition. Nevertheless, the specificities for the 2 definitions were 89% or higher with the lower cutoffs and 98% or higher with the higher cutoffs. The POW cutoff of 20%, which has been used in Japan to define obesity, had higher sensitivity but lower specificity than the corresponding cutoffs derived from the IOTF-BMI definition. The BMI and POW ROC curves largely overlapped (Figures 1 and 2); however, for the 10-year-old girls, the AUC of the BMI curve (≥2, ≥3 risks) was significantly larger than that of the POW curve (*P* < 0.001 and *P* = 0.013, respectively). This difference was also observed for other risk factor cutoffs (*P* < 0.05).

**Table 4.** Sensitivities and specificities of the 2 definitions of overweight

	Lower cutoff (POW, 20%; IOTF, 25 kg/m^2^)			Higher cutoff (POW, 50%; IOTF, 30 kg/m^2^)		
		
	*n* (%obese subjects)	Sensitivity (%)	Specificity (%)	*n* (%obese subjects)	Sensitivity (%)	Specificity (%)
≥1 risk factor, *n* = 2193						
POW	283 (86)	13	97	28 (97)	1	100
IOTF	363 (83)	17	95	63 (91)	3	100

≥2 risk factors, *n* = 889						
POW	189 (57)	21	95	23 (79)	3	100
IOTF	228 (52)	26	93	48 (70)	5	99

≥3 risk factors, *n* = 196						
POW	66 (20)	34	93	12 (41)	6	100
IOTF	81 (18)	41	90	22 (32)	11	99

4 risk factors, *n* = 16						
POW	10 (3)	63	92	1 (3)	6	99
IOTF	12 (3)	75	89	3 (4)	19	98

POW, percentage overweight; IOTF-BMI, International Obesity Taskforce definition, using the BMI of an 18-year-old as reference.

## DISCUSSION

In the present study, the AUC of the POW ROC curve mostly overlapped that of the BMI ROC curve, except in the case of the 10-year-old girls. Although the POW-based cutoff had the lower sensitivity, it had higher specificity for risk factor screening. The POW cutoff of 20%, which was originally used in Japan to define obesity, had lower specificity than the IOTF-BMI cutoff. Lower specificity results in a higher rate of false positives. Children and adolescents classified as obese have psychosocial burdens, and reliable intervention methods have not been established for overweight children and adolescents. Moreover, the extent to which risk factors detected in children and adolescents are associated with future health status is unknown. However, the BMI cutoff values for adult obesity are lower in Japan than in other countries^18^ because health risks related to obesity appear to be present at lower BMI values in Asians.^19^ Although a POW of 20% or more is the highest cutoff for obesity in Japan, no projects have been undertaken to make children and adolescents in this category more aware of obesity-related health risks or to encourage lifestyle changes. The results of the present study suggest that BMI-based cutoffs may be as effective as POW-based cutoffs for screening obesity-related risk factors among Japanese children and adolescents, although POW-cutoffs are useful in that they remain constant regardless of the age of the subject being screened.

Both Asian children^20^ and adults^21^^,^^22^ have higher body fat percentages at a given BMI than do whites. As compared to BMI, waist circumference is considered a better indicator of adiposity in adults.^23^^,^^24^ In studies of obese children, waist circumference has been identified as a potential predictor of risk.^25^^,^^26^ However, large-scale cross-sectional^16^ and longitudinal^15^ studies have revealed that waist circumference and BMI differ negligibly among children and adolescents. Furthermore, BMI is a stronger predictor of hypertension than is the waist-hip ratio.^3^ Because BMI is determined by simple anthropometric measurements, which are carried out annually in schools across Japan in accordance with the School Health Law, it can be used as a straightforward screening tool as part of a comprehensive public health strategy.

When children are screened for lifestyle diseases, postprandial blood samples are often preferred to fasting blood samples, because requiring school-aged children to fast before giving blood may reduce their physical stamina.^13^ In the present study, cholesterol levels among nonfasting subjects were lower than those of fasting subjects, and the difference was greater than that seen for plasma glucose levels among the same blood samples. This result helps explain why hematocrit and hemoglobin levels were higher in the fasting samples than in the postprandial samples (*P* < 0.01; data not shown). As a primary screening tool, body weight evaluation is therefore preferable to invasive procedures such as blood testing.

In the present study, dyscholesterolemia was not associated with the weight status of female participants; this finding was consistent with the results of a previous study of adolescents.^1^^,^^17^^,^^27^ Furthermore, among the male participants, hyperglycemia was not associated with being overweight or obese. The incidence of diabetes mellitus is relatively high among Japanese with low BMI values,^28^ a fact that may be attributable to the presence of decreased insulin secretion, rather than obesity-related insulin resistance.^29^ Thus, body weight monitoring may not allow for complete risk-factor screening. In such cases, a family history (with identification of underlying genetic factors) may be the optimal screening tool.^30^

The present study has several limitations. Age-specific cutoffs for risks are unsatisfactory because cholesterol levels decrease with age, while blood pressure levels increase. Height-specific blood pressure cutoffs should be used, in accordance with the National Institutes of Health guidelines^31^; however, height references differ between races. We rounded off the participants’ ages, as required for the IOTF and POW definitions. Given the age intervals used for these definitions, the prevalence of overweight/obesity may be underestimated.^32^ Although children exhibit a growth spurt around the age of our study subjects,^33^ we could not obtain any data regarding the onset of puberty among our subjects. In addition, the study population that was examined in the present report may not have been representative of the overall Japanese population. However, a 2006 study of children and adolescents with ages similar to the participants of the present study revealed that the mean prevalence of overweight individuals (as per the POW definition) was 8.6% to 11.7% (range, 4.3%–19.6%) across 47 Japanese prefectures,^34^ which was similar to that noted in the present study. A further limitation of the present study is that some blood pressure measurements might have been erroneous due to the use of automatic monitors. In addition, the cross-sectional design is not adequate for predicting future obesity-related diseases. Additional risk factors, such as serum transaminase and insulin levels, should be evaluated to predict subsequent life health status.^25^ Finally, the results cannot be applied to age groups other than those examined in the present study. The ROC curve of children younger than 10 years may differ from those of older children, as was the case for the curves of the younger and older children in the present study.

In summary, both BMI-based and POW-based cutoffs are useful for screening Japanese children and adolescents for risk factors, although POW-based cutoffs may be slightly less useful. The community-based design, relatively large sample size, and low dropout rate of the present study strengthen the validity of the results. An important finding of the present study is that individuals may be wrongly classified with risk factors, especially dyscholesterolemia, if postprandial blood samples are used. We did not measure waist circumference, which is often used as a diagnostic criterion for metabolic syndrome.^35^^,^^36^ It remains to be determined whether this parameter is superior to BMI or POW as a screening tool for risk factors among Japanese children and adolescents.
